# Anxiolytic and Anxiogenic? How the Transcription Factor MEF2 Might Explain the Manifold Behavioral Effects of Oxytocin

**DOI:** 10.3389/fendo.2020.00186

**Published:** 2020-04-08

**Authors:** Benjamin Jurek, Magdalena Meyer

**Affiliations:** Department of Behavioural and Molecular Neurobiology, Institute of Zoology, University of Regensburg, Regensburg, Germany

**Keywords:** oxytocin, salience, MEF2, anxiety, stress, cellular morphology

## Abstract

The neuromodulator oxytocin, since its first synthesis by du Vigneaud in 1953, has mainly been associated with beneficial physiological effects, as well as positive social and emotional behaviors. This overall positive picture of oxytocin as the “love-, cuddle-, or bonding-hormone” has repeatedly been challenged since then. Oxytocin-induced effects that would be perceived as negative by the individual, such as increased anxiety or potentiation of stress-induced ACTH release, as well as the regulation of negative approach-related emotions, such as envy and schadenfreude (gloating) have been described. The general consent is that oxytocin, instead of acting unidirectional, induces changes in the salience network to shift the emphasis of emotional contexts, and therefore can, e.g., produce both anxiolytic as well as anxiogenic behavioral outcomes. However, the underlying mechanisms leading to alterations in the salience network are still unclear. With the aim to understand the manifold effects of oxytocin on a cellular/molecular level, a set of oxytocin receptor-coupled signaling cascades and downstream effectors regulating transcription and translation has been identified. Those oxytocin-driven effectors, such as MEF2 and CREB, are known modulators of the neuronal and glial cytoarchitecture. We hypothesize that, by determining cellular morphology and connectivity, MEF2 is one of the key factors that might contribute to the diverse behavioral effects of oxytocin.

## Introduction

The neuropeptide oxytocin is mainly produced in the hypothalamic paraventricular (PVN) and supraoptic nuclei (SON), it is released from magnocellular cells by axonal and somatodendritic release, and into the periphery via axonal projections (1). Peripheral axonal release into the blood stream and central dendritic release can occur in an orchestrated or independent manner (2–4) and produces a variety of behavioral effects, ranging from anxiety, fear conditioning and fear extinction, social exploration and recognition to aggressive behavior (5). After taking differences in dosing, timing, species, or central vs. peripheral release into account, we are still left with some puzzling inconsistencies regarding the behavioral effects of oxytocin [for review see (1)]. The concept to explain and interpret the behavioral effects of oxytocin is centered around a change in salience of emotional contexts (6–8), rather than unidirectional influences on certain types of behavior (e.g., anxiety). Our aim is to establish a coherent mechanistic understanding of the intracellular processes that underlie this change in emotional salience and therefore the effects on opposing behavioral outcomes.

## Alleviating Behavioral Effects of Oxytocin

In rodents, the level of anxiety can be assessed by testing the exploratory drive vs. the fear of open and brightly lit spaces, as it is done in the elevated plus maze (EPM) or Light Dark Box (LDB). In previous publications, we have established that an infusion of synthetic oxytocin directly into the PVN of male and female rats reduces anxiety-like behavior for at least 4 h, i.e., increases the time spent in the open arms or lit compartment of the EPM and LDB, respectively (9–12). The endogenous release of oxytocin within the PVN can be triggered in male and female rats by various stimuli (13, 14). For instance, mating leads to a similar anxiolysis as observed with synthetic oxytocin infusions, indicating the behavioral valence of synthetic oxytocin (15, 16). In female prairie voles, intra-PVN infusions of oxytocin reduce anxiety-like behavior by activating local GABAergic neurons, which dampen the stress-induced activity of CRF-positive neurons and subsequent corticosterone release (17). As oxytocin receptors are also expressed on CRF-positive neurons (18), it not only acts via GABAergic inhibition, but also relies on direct oxytocin receptor-coupled modulation of stress-induced CRF transcription in the PVN of male rats as well (19). The interplay between oxytocin and the CRF system is not restricted to the PVN, as oxytocin receptor interneurons in the medial prefrontal cortex are responsible for anxiolysis in male mice, whereas in female mice they act pro-social by GABAergic inhibition of CRF receptor 1 expressing neurons (20, 21).

In a similar manner, infusions into the central amygdala reduce anxiety as well (22), and evoked release of endogenous oxytocin attenuates freezing in fear-conditioned mice (23) by acting on a local GABAergic inhibitory output circuit (24, 25). The type of fear response displayed, i.e., either startle response or active escape, is orchestrated by glutamatergic projections from the basolateral amygdala to oxytocin receptor positive cells in the central amygdala of mice and humans (26). Oxytocin also attenuates social fear expression in male, virgin and lactating female mice (27, 28), and cued fear extinction in male mice (29).

Those positive “dampening” or “alleviating” effects of oxytocin have been well-established in literature, so much, that a comprehensive list of all studies would be beyond the scope of this review [for extended discussion see (1)]. However, not all findings are in line with such a “positive” image of oxytocin. Depending on the context, oxytocin can produce opposing, more adverse or negative effects on behavior.

## Adverse Behavioral Effects of Oxytocin

However, well-described the alleviating effects of oxytocin might be, potentiating effects of oxytocin on the stress response and increased anxiety have to be reflected to generate a comprehensive understanding. For instance, when peripheral oxytocin levels are increased chronically by subcutaneous infusion in male rats over the course of 14 days, ACTH and corticosterone levels, and adrenal weights are increased, indicating a potentiating effect of oxytocin on the HPA axis, and therefore the stress response (30). In addition, single subcutaneous injections transiently increase plasma ACTH and corticosterone levels in male rats (31), but decrease corticosterone levels 6 h after the injection without affecting ACTH levels. In an *ex vivo* setting, i.e., isolated hemipituitaries from male rats, oxytocin potentiated the CRF-induced ACTH release (32). In an *in vivo* setting, peripheral oxytocin secretion is modestly influenced by the activated HPA axis, i.e., plasma corticosterone levels; however, corticosterone levels amplified stress-induced oxytocin release within the PVN (33). Those data delineate oxytocin as a modulator of activated systems like the HPA axis instead of acting as a solitary driving force. However, a recent study conducted in wild chimpanzees failed to associate oxytocinergic system activity with increased stress and aggression during out-group conflict (34), which is seemingly in contrast to predictions made by the social salience hypothesis of oxytocin. However, whether urinary oxytocin and cortisol levels reflect central or plasma concentrations has yet to be determined, as a random discrepancy between those body-fluids might be the cause for the failed association.

In addition, timing and dosage seem to be key aspects that orchestrate the functional outcome. For instance, in a mouse model of chronic oxytocin infusion via osmotic minipumps, a low dose of chronic oxytocin (1 ng/0.2 μl/h) alleviated the effects of chronic stress, such as thymus atrophy, adrenal hypertrophy and decreased *in vitro* adrenal ACTH sensitivity; whereas a high dose (10 ng/0.2 μl/h) increased anxiety-like behavior in male mice (35). The increased anxiety was concomitant with a decreased expression of oxytocin receptors in the septum, likely as part of a negative feedback regulation, indicating this region as one important regulatory region for oxytocin-driven anxiety. However, the expression of fear in socially defeated male mice is positively associated with the expression level of oxytocin receptors and its coupling to the MAP kinase pathway in the lateral septum (36). When knocked down, the level of fear displayed by socially defeated mice toward their defeater was diminished, compared to mice where oxytocin receptors were overexpressed. The authors conclude that oxytocin does not have a unidirectional influence on anxiety, but rather changes the salience or valence of an emotional context (36). Another study revealed a full rescue of socially transmitted fear in unfamiliar male mice together with an enhanced cellular activity within the anterior cingulate cortex after acute intranasal oxytocin administration (37). To the contrary, the same study also investigated the effects of chronic oxytocin administration, which led to long-term facilitation of observational fear. Interestingly, none of these manipulations affected fear acquired as a result of direct experience with the stressor, but only socially transmitted fear. Hence, these results emphasize the role of oxytocin in context-dependent empathy.

Effects on empathy and context-dependent social cues have also been studied in human probands that received intranasal oxytocin. Those studies found increased aggression toward game partners in the “social orientation paradigm” (38), increased envy and schadenfreude or gloating in a game of chance (39), and even increased anxiety, indicated by an enhanced startle response after unpredictable threats (40).

**Figure 1:**
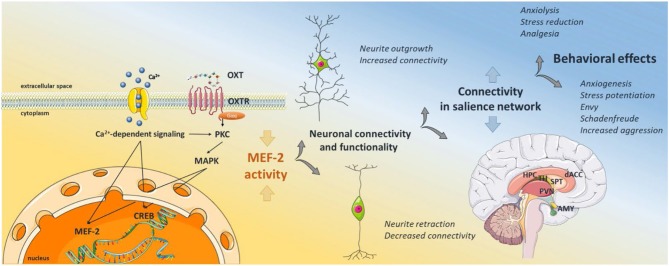
Graphical Abstract of the proposed signaling scheme. Oxytocin binds its receptor, which transactivates different types of calcium channels, leading to calcium influx from the extracellular space. Calcium activates protein kinase C, and subsequent MAPK pathway activation, i.e., MEK1/2-ERK1/2, which translocates to the nucleus and activates the transcription factor CREB. Calcium also activates the calcium-dependent phosphatase calcineurin, which dephosphorylates and therefore activates MEF2. Active MEF2 increases gene transcription of negative regulators of synaptic structures, causing decreased neurite outgrowth, synapse number, and hypoconnectivity between neighboring neurons. As oxytocin receptor positive neurons are located within brain regions of the salience network, MEF2 activity regulates the connectivity within those regions, dampening or increasing the activity in the whole network. Depending on the emotional context of external stimuli, those changes in the salience network can cause alleviating or adverse behavioral responses. The schematic art pieces used in this figure were provided by Servier Medical art (http://servier.com/Powerpoint-image-bank). Servier Medical Art by Servier is licensed under a Creative Commons Attribution 3.0 Unported License.

Most of the above-mentioned studies interpret their data according to the salience hypothesis stating that oxytocin increases the perception of social stimuli dependent on the context, instead of acting unidirectional on any behavior. Thus, if oxytocin is not a pure anxiolytic, analgesic, or anti-stress hormone, but rather shifts the salience of an emotional context, changes in the activity of the salience network must be detectable (see Figure 1).

## Oxytocin and the Salience Network

In order to understand the multitude of behavioral effects of oxytocin, a theoretical framework has been proposed that includes a prominent role for oxytocin-dopamine-interactions in regulating the salience of social cues (41). For instance, a recent study conducted in titi monkeys found increased anxiety and a trend toward increased glucose uptake in regions of the salience network. Specifically, glucose uptake tended to increase in the SON and caudate nucleus, in male animals treated chronically with intranasal oxytocin from 12 to 18 months of age (42). In addition to the SON and caudate nucleus, the salience network consists of other brain regions that have already been associated with oxytocinergic effects and that express the oxytocin receptor, like the anterior cingulate cortex, lateral septum, striatum, medial prefrontal cortex, hippocampus, amygdala, nucleus accumbens, and PVN (42). The significance of the salience network for psychiatric disorders has been shown by a very comprehensive meta-analysis that included more than 7,000 individuals with psychiatric diagnoses ranging from depression, bipolar disorder, schizophrenia, or obsessive-compulsive disorder, to anxiety disorders (43). In each of those disorders, changes in myelin protein expression (44, 45), decreased gray and white matter volume (46), and loss of connectivity have been detected in core regions of the salience network, like the anterior cingulate cortex or medial prefrontal cortex, leading to impairments of cognitive control, which has been proposed as a diagnostic feature across the above-mentioned psychiatric disorders (47).

Besides alterations in oligodendrocyte-based myelin formation or damage, reduced gray matter volume and connectivity can be caused either by morphological changes of neurons (i.e., increased or decreased synapse formation) or by reduced neuronal survival rates. Consequentially, factors that influence those two aspects, and are triggered by oxytocin could be the sought after molecular substrates of the behavioral effects of oxytocin. In search for those factors, we investigated the signaling cascades coupled to the oxytocin receptor.

## Oxytocin and MEF2

A first clue can be drawn from the apparent universal involvement of the MAP kinase pathway in the above-described contexts, e.g., the fear enhancing effects in the lateral septum (36), or the anxiolytic and anti-stress effects in the PVN (9, 10, 19). Potential targets of the MEK1/2-ERK1/2 pathway are on the one hand the transcription factor CREB, which is involved in oxytocin-induced spatial memory formation (48) and CRF gene transcription (19). On the other hand, the transcription factor myocyte enhancer factor 2 (MEF2), which has been shown to be directly activated following oxytocin receptor activation (49, 50) via the calcium-dependent phosphatase calcineurin (51). MEF2 is a main regulator of neuronal morphology, survival, connectivity, plasticity, and metaplasticity (52–54). CREB and MEF2 can act independently, but have also been shown to bind in concert to the synaptic response element (SARE), an enhancer sequence found upstream of many neuronal plasticity-related genes (55, 56). While we have identified a MEF2 binding sequence in the oxytocin receptor promoter with *in silico* prediction tools, there is no recent evidence available, whether activated MEF2 alters the transcription of the *Oxtr* gene. Decreased MEF2 levels and increased phosphorylation levels, i.e., decreased gene transcription, have been found in mice that underwent fear conditioning and spatial memory tasks. In contrast, increasing MEF2 levels and dephosphorylation prevented the formation of spatial memory and associated increase in spine density. These findings suggest that MEF2-mediated transcription constrains memory formation by interfering with neuronal plasticity (57).

## MEF2 and Neuronal Connectivity

Neuronal plasticity and connectivity is governed by the ability of the neurons to adapt their cellular morphology to the momentary requirements, i.e., induce neurite outgrowth and retraction. Both factors, CREB and MEF2 have been shown to actively regulate neurite outgrowth (58, 59) and thus influence the formation of synapses (60, 61). Those oxytocin-induced cellular effects can depend on MEF2 expression levels as the transcription factor is known to be a regulator of metaplasticity, i.e., it determines how neurons respond to stimuli by shifting plasticity thresholds (54). Moreover, mutated MEF2 has been associated with Rett-like syndrome (62, 63) and autism spectrum disorder (64–66). Recent publications indicate a connection between dysregulated synapse number, i.e., hyperconnectivity, and symptoms of autism spectrum disorder (67); moreover, lower plasma oxytocin levels have been reported in children with autism and social impairments (68, 69). Those impairments and low plasma oxytocin levels might be associated with an altered composition of the gut microbiome (70), a process that also impacts MEF2 phosphorylation (71), and neuronal plasticity (72). A current study revealed that in male patients with autism spectrum disorder higher levels of endogenous salivary oxytocin are associated with lower degrees of functional coupling between the amygdala and hippocampus (73). Notably, a single dose of intranasal oxytocin induced a further reduction in the degree of functional connectivity between those two regions. Those data suggest that abnormal oxytocin synthesis and/or intracerebral release could be the cause for the observed connectivity differences in autistic patients. In support of this hypothesis, we and others have recently shown that oxytocin modulates neuronal morphology and synapse formation in dependence of MEF2A, calcium signaling, and the MAPK pathway (50, 74–78).

## MEF2-Driven Mechanisms Affecting Neuronal Connectivity

In order to elucidate the underlying signaling pathways explaining the manifold behavioral effects of oxytocin, we have gathered evidence for a central role of the transcription factor MEF2. The activity-dependent influence on neuronal plasticity, termed metaplasticity, is one key characteristic trait of MEF2 (54, 79), and could explain the multitude of behavioral effects of oxytocin, by altering the connectivity, and therefore activity, between neurons of brain regions that are part of the salience network (e.g., within the mPFC, hypothalamus, or amygdala). Altered activity within those oxytocin-sensitive brain regions dampens or stimulates adjacent regions (80, 81), and therefore the whole network. The molecular machinery that determines MEF2 activity, and therefore causes this plasticity, has been investigated in detail. MEF2 contains several different phosphorylation sites, acting either inhibitory, or stimulatory on gene transcription (52). Two different calcium-dependent pathways have been described: (1) Class II HDACs are bound to MEF2, acting as permanent inhibitors. Upon calcium influx, these HDACs are phosphorylated by calcium/calmodulin activated kinase (CaMK) and exported out of the nucleus, relieving MEF2 repression and allowing for the activation of MEF2-dependent transcription (58). (2) Calcium influx leads to the dephosphorylation of MEF2 proteins at serine 408, via the calcium-dependent phosphatase calcineurin (60, 82). This dephosphorylation promotes a switch from sumoylation to acetylation at lysine 403, which leads to inhibition of dendritic claw differentiation (82). The question remains how activated (acetylated and dephosphorylated) MEF2 regulates synapse number, and therefore connectivity between neurons. Several possible mechanisms have been described: first, activated MEF2 promotes the sumoylation and subsequent degradation of a synaptic scaffolding protein, the postsynaptic density protein 95 (PSD-95) (83). Second, the transcription of negative regulators of synapse development, arc and synGAP, is stimulated by activated MEF2, leading to a reduced excitatory synapse number in hippocampal neurons (60). And lastly, MEF2 closely interacts with another regulator of neuronal connectivity, the mitochondria (79). We have shown that MEF2 expression modulates the basic mitochondrial functions like maximal respiration, spare respiratory capacity, and ATP production in neurons by means of a CRISPR-Cas-generated MEF2A functional knockout cell line (51). Those mitochondrial mechanisms are known to regulate synaptic strength (79). Since mitochondria have their own genome, transcription factors like MEF2 are able to induce mitochondrial gene transcription in addition to nuclear transcription. Indeed, MEF2 promotes the expression of the mitochondrial gene ND6, which is essential for the function of the oxidative phosphorylation system (84), which regulates the production of ROS. Release of ROS activates the pro-apoptotic caspase-3, which induces the breakdown of PSD-95, thereby weakening synaptic strength (79).

Taken together, the above-described effects of MEF2 on synaptic strength can be bidirectional, as MEF2 can either stimulate or inhibit gene transcription (85). Oxytocin receptor signaling activates MEF2 in a context-dependent manner and thereby induces morphological changes, e.g., neurite retraction (50). Morphological changes in turn determine the synaptic strength and therefore connectivity between oxytocin receptor positive neurons (76). Oxytocin-sensitive neurons are located in brain regions that are part of the salience network (80), which has been shown to be responsive to oxytocin (42) and modulates anxiety-like behavior or the stress response (86, 87).

## Conclusion

In conclusion, oxytocin exerts its behavioral effects not in a direct manner, but rather acts modulatory on the activated salience network, to alter the emotional context of a secondary stimulus and produce a variety of context-dependent behavioral outcomes, including anxiolysis and anxiogenesis and increased or reduced stress response. We hypothesize that MEF2 is one of the key elements enabling this behavioral variability by modulating the connectivity of oxytocin receptor-positive neurons within a brain region and as result of this, potentially affecting the salience network as a whole.

## Author Contributions

BJ and MM contributed equally to the manuscript.

### Conflict of Interest

The authors declare that the research was conducted in the absence of any commercial or financial relationships that could be construed as a potential conflict of interest.

## References

[1] Jurek B Neumann ID. The oxytocin receptor: from intracellular signaling to behavior. Physiol Rev. (2018) 98:1805–908. 10.1152/physrev.00031.2017 29897293

[2] Ludwig M Leng G Dendritic peptide release and peptide-dependent behaviors. Nat Rev Neurosci. (2006) 7:126–36. 10.1038/nrn1845 16429122

[3] Neumann ID. Stimuli and consequences of dendritic release of oxytocin within the brain. Biochem Soc Trans. (2007) 35(Pt 5):1252–7. 10.1042/BST0351252 17956324

[4] Neumann ID Landgraf R. Balance of brain oxytocin and vasopressin: implications for anxiety, depression, and social behaviors. Trends Neurosci. (2012) 35:649–59. 10.1016/j.tins.2012.08.004 22974560

[5] Meyer-Lindenberg A Domes G Kirsch P Heinrichs M. Oxytocin and vasopressin in the human brain: social neuropeptides for translational medicine. Nat Rev Neurosci. (2011) 12:524–38. 10.1038/nrn3044 21852800

[6] Di Simplicio M Massey-Chase R Cowen PJ Harmer CJ. Oxytocin enhances processing of positive versus negative emotional information in healthy male volunteers. J Psychopharmacol. (2009) 23:241–8. 10.1177/0269881108095705 18801829

[7] Quintana DS Westlye LT Hope S Naerland T Elvsashagen T Dorum E. Dose-dependent social-cognitive effects of intranasal oxytocin delivered with novel Breath Powered device in adults with autism spectrum disorder: a randomized placebo-controlled double-blind crossover trial. Transl Psychiatry. (2017) 7:e1136. 10.1038/tp.2017.103 28534875 PMC5584522

[8] Duque-Wilckens N Steinman MQ Busnelli M Chini B Yokoyama S Pham M. Oxytocin receptors in the anteromedial bed nucleus of the stria terminalis promote stress-induced social avoidance in female california mice. Biol Psychiatry. (2018) 83:203–13. 10.1016/j.biopsych.2017.08.024 29066224 PMC5743604

[9] Blume A Bosch OJ Miklos S Torner L Wales L Waldherr M. Oxytocin reduces anxiety via ERK1/2 activation: local effect within the rat hypothalamic paraventricular nucleus. Eur J Neurosci. (2008) 27:1947–56. 10.1111/j.1460-9568.2008.06184.x 18412615

[10] Jurek B Slattery DA Maloumby R Hillerer K Koszinowski S Neumann ID. Differential contribution of hypothalamic MAPK activity to anxiety-like behaviour in virgin and lactating rats. PLoS ONE. (2012) 7:e37060. 10.1371/journal.pone.0037060 22615888 PMC3355176

[11] van den Burg EH Stindl J Grund T Neumann ID Strauss O. Oxytocin stimulates extracellular Ca2+ influx through TRPV2 channels in hypothalamic neurons to exert its anxiolytic effects. Neuropsychopharmacology. (2015) 40:2938–47. 10.1038/npp.2015.147 26013963 PMC4864629

[12] Martinetz S Meinung CP Jurek B von Schack D van den Burg EH Slattery DA. *De Novo* protein synthesis mediated by the eukaryotic elongation factor 2 is required for the anxiolytic effect of oxytocin. Biol Psychiatry. (2019) 85:802–11. 10.1016/j.biopsych.2019.01.010 30826070

[13] Landgraf R Neumann ID. Vasopressin and oxytocin release within the brain: a dynamic concept of multiple and variable modes of neuropeptide communication. Front Neuroendocrinol. (2004) 25:150–76. 10.1016/j.yfrne.2004.05.001 15589267

[14] Neumann ID. Brain oxytocin: a key regulator of emotional and social behaviours in both females and males. J Neuroendocrinol. (2008) 20:858–65. 10.1111/j.1365-2826.2008.01726.x 18601710

[15] Waldherr M Neumann ID. Centrally released oxytocin mediates mating-induced anxiolysis in male rats. Proc Natl Acad Sci USA. (2007) 104:16681–4. 10.1073/pnas.0705860104 17925443 PMC2034242

[16] Nyuyki KD Waldherr M Baeuml S Neumann ID. Yes, I am ready now: differential effects of paced versus unpaced mating on anxiety and central oxytocin release in female rats. PLoS ONE. (2011) 6:e23599. 10.1371/journal.pone.0023599 21858181 PMC3156771

[17] Smith AS Tabbaa M Lei K Eastham P Butler MJ Linton L. Local oxytocin tempers anxiety by activating GABAA receptors in the hypothalamic paraventricular nucleus. Psychoneuroendocrinology. (2016) 63:50–8. 10.1016/j.psyneuen.2015.09.017 26415118 PMC4695278

[18] Dabrowska J Hazra R Guo JD Dewitt S Rainnie DG Central CRF neurons are not created equal: phenotypic differences in CRF-containing neurons of the rat paraventricular hypothalamus and the bed nucleus of the stria terminalis. Front Neurosci. (2013) 7:156 10.3389/fnins.2013.00156 24009552 PMC3757458

[19] Jurek B Slattery DA Hiraoka Y Liu Y Nishimori K Aguilera G. oxytocin regulates stress-induced Crf gene transcription through CREB-regulated transcription coactivator 3. J Neurosci. (2015) 35:12248–60. 10.1523/JNEUROSCI.1345-14.2015 26338335 PMC4556790

[20] Nakajima M Gorlich A Heintz N. Oxytocin modulates female sociosexual behavior through a specific class of prefrontal cortical interneurons. Cell. (2014) 159:295–305. 10.1016/j.cell.2014.09.020 25303526 PMC4206218

[21] Li K Nakajima M Ibanez-Tallon I Heintz N. A cortical circuit for sexually dimorphic oxytocin-dependent anxiety behaviors. Cell. (2016) 167:60–72 e11. 10.1016/j.cell.2016.08.067 27641503 PMC5220951

[22] Okimoto N Bosch OJ Slattery DA Pflaum K Matsushita H Wei FY. RGS2 mediates the anxiolytic effect of oxytocin. Brain Res. (2012) 1453:26–33. 10.1016/j.brainres.2012.03.012 22459044

[23] Knobloch HS Charlet A Hoffmann LC Eliava M Khrulev S Cetin AH. Evoked axonal oxytocin release in the central amygdala attenuates fear response. Neuron. (2012) 73:553–66. 10.1016/j.neuron.2011.11.030 22325206

[24] Huber D Veinante P Stoop R. Vasopressin and oxytocin excite distinct neuronal populations in the central amygdala. Science. (2005) 308:245–8. 10.1126/science.1105636 15821089

[25] Viviani D Charlet A van den Burg E Robinet C Hurni N Abatis M. Oxytocin selectively gates fear responses through distinct outputs from the central amygdala. Science. (2011) 333:104–7. 10.1126/science.1201043 21719680

[26] Terburg D Scheggia D Triana Del Rio R Klumpers F Ciobanu AC Morgan B. The basolateral amygdala is essential for rapid escape: a human and rodent study. Cell. (2018) 175:723–35 e716. 10.1016/j.cell.2018.09.028 30340041 PMC6198024

[27] Zoicas I Slattery DA Neumann ID. Brain oxytocin in social fear conditioning and its extinction: involvement of the lateral septum. Neuropsychopharmacology. (2014) 39:3027–35. 10.1038/npp.2014.156 24964815 PMC4229574

[28] Menon R Grund T Zoicas I Althammer F Fiedler D Biermeier V. Oxytocin signaling in the lateral septum prevents social fear during lactation. Curr Biol. (2018) 28:1066–78 e1066. 10.1016/j.cub.2018.02.044 29551417

[29] Toth I Neumann ID Slattery DA. Central administration of oxytocin receptor ligands affects cued fear extinction in rats and mice in a timepoint-dependent manner. Psychopharmacology. (2012) 223:149–58. 10.1007/s00213-012-2702-4 22526533

[30] Ondrejcakova M Bakos J Garafova A Kovacs L Kvetnansky R Jezova D. Neuroendocrine and cardiovascular parameters during simulation of stress-induced rise in circulating oxytocin in the rat. Stress. (2010) 13:314–22. 10.3109/10253891003596822 20536333

[31] Petersson M Hulting AL Uvnas-Moberg K. Oxytocin causes a sustained decrease in plasma levels of corticosterone in rats. Neurosci Lett. (1999) 264:41–4. 10.1016/S0304-3940(99)00159-7 10320009

[32] Gibbs DM Vale W Rivier J Yen SS Oxytocin potentiates the ACTH-releasing activity of CRF(41) but not vasopressin. Life Sci. (1984) 34:2245–9 10.1016/0024-3205(84)90212-1 6328161

[33] Torner L Plotsky PM Neumann ID de Jong TR. Forced swimming-induced oxytocin release into blood and brain: effects of adrenalectomy and corticosterone treatment. Psychoneuroendocrinology. (2017) 77:165–74. 10.1016/j.psyneuen.2016.12.006 28064086

[34] Samuni L Preis A Deschner T Wittig RM Crockford C. Cortisol and oxytocin show independent activity during chimpanzee intergroup conflict. Psychoneuroendocrinology. (2019) 104:165–73. 10.1016/j.psyneuen.2019.02.007 30851601

[35] Peters S Slattery DA Uschold-Schmidt N Reber SO Neumann ID. Dose-dependent effects of chronic central infusion of oxytocin on anxiety, oxytocin receptor binding and stress-related parameters in mice. Psychoneuroendocrinology. (2014) 42:225–36. 10.1016/j.psyneuen.2014.01.021 24636519

[36] Guzman YF Tronson NC Jovasevic V Sato K Guedea AL Mizukami H. Fear-enhancing effects of septal oxytocin receptors. Nat Neurosci. (2013) 16:1185–7. 10.1038/nn.3465 23872596 PMC3758455

[37] Pisansky MT Hanson LR Gottesman II Gewirtz JC. Oxytocin enhances observational fear in mice. Nat Commun. (2017) 8:2102. 10.1038/s41467-017-02279-5 29235461 PMC5727393

[38] Ne‘eman R Perach-Barzilay N Fischer-Shofty M Atias A Shamay-Tsoory SG. Intranasal administration of oxytocin increases human aggressive behavior. Horm Behav. (2016) 80:125–31. 10.1016/j.yhbeh.2016.01.015 26862988

[39] Shamay-Tsoory SG Fischer M Dvash J Harari H Perach-Bloom N Levkovitz Y. Intranasal administration of oxytocin increases envy and schadenfreude (gloating). Biol Psychiatry. (2009) 66:864–70. 10.1016/j.biopsych.2009.06.009 19640508

[40] Grillon C Krimsky M Charney DR Vytal K Ernst M Cornwell B. Oxytocin increases anxiety to unpredictable threat. Mol Psychiatry. (2013) 18:958–60. 10.1038/mp.2012.156 23147382 PMC3930442

[41] Shamay-Tsoory SG Abu-Akel A. The social salience hypothesis of oxytocin. Biol Psychiatry. (2016) 79:194–202. 10.1016/j.biopsych.2015.07.020 26321019

[42] Arias Del Razo R Berger T Conley AJ Freeman SM Goetze LR Jacob S. Effects of chronic intranasal oxytocin on behavior and cerebral glucose uptake in juvenile titi monkeys. Psychoneuroendocrinology. (2019) 113:104494. 10.1016/j.psyneuen.2019.104494 31862614 PMC7909742

[43] Goodkind M Eickhoff SB Oathes DJ Jiang Y Chang A Jones-Hagata LB. Identification of a common neurobiological substrate for mental illness. JAMA Psychiatry. (2015) 72:305–15. 10.1001/jamapsychiatry.2014.2206 25651064 PMC4791058

[44] Honer WG Falkai P Chen C Arango V Mann JJ Dwork AJ. Synaptic and plasticity-associated proteins in anterior frontal cortex in severe mental illness. Neuroscience. (1999) 91:1247–55. 10.1016/S0306-4522(98)00679-4 10391432

[45] Nave KA Ehrenreich H. Myelination and oligodendrocyte functions in psychiatric diseases. JAMA Psychiatry. (2014) 71:582–4. 10.1001/jamapsychiatry.2014.189 24671770

[46] Bakhtiari R Zurcher NR Rogier O Russo B Hippolyte L Granziera C. Differences in white matter reflect atypical developmental trajectory in autism: A Tract-based Spatial Statistics study. Neuroimage Clin. (2012) 1:48–56. 10.1016/j.nicl.2012.09.001 24179736 PMC3757732

[47] Peters SK Dunlop K Downar J. Cortico-striatal-thalamic loop circuits of the salience network: a central pathway in psychiatric disease and treatment. Front Syst Neurosci. (2016) 10:104. 10.3389/fnsys.2016.00104 28082874 PMC5187454

[48] Tomizawa K Iga N Lu YF Moriwaki A Matsushita M Li ST. Oxytocin improves long-lasting spatial memory during motherhood through MAP kinase cascade. Nat Neurosci. (2003) 6:384–90. 10.1038/nn1023 12598900

[49] Devost D Wrzal P Zingg HH. Oxytocin receptor signalling. Prog Brain Res. (2008) 170:167–76. 10.1016/S0079-6123(08)00415-9 18655881

[50] Meyer M Berger I Winter J Jurek B. Oxytocin alters the morphology of hypothalamic neurons via the transcription factor myocyte enhancer factor 2A (MEF-2A). Mol Cell Endocrinol. (2018) 477:156–62. 10.1016/j.mce.2018.06.013 29928931

[51] Meyer M Kuffner K Winter J Neumann ID Wetzel CH Jurek B. Myocyte enhancer factor 2A (MEF2A) defines oxytocin-induced morphological effects and regulates mitochondrial function in neurons. Int J Mol Sci. (2020) 21:2200. 10.3390/ijms21062200 32209973 PMC7139413

[52] Potthoff MJ Olson EN. MEF2: a central regulator of diverse developmental programs. Development. (2007) 134:4131–40. 10.1242/dev.008367 17959722

[53] Akhtar MW Kim MS Adachi M Morris MJ Qi X Richardson JA. In vivo analysis of MEF2 transcription factors in synapse regulation and neuronal survival. PLoS ONE. (2012) 7:e34863. 10.1371/journal.pone.0034863 22496871 PMC3322166

[54] Chen SX Cherry A Tari PK Podgorski K Kwong YK Haas K. The transcription factor MEF2 directs developmental visually driven functional and structural metaplasticity. Cell. (2012) 151:41–55. 10.1016/j.cell.2012.08.028 23021214

[55] Rodriguez-Tornos FM San Aniceto I Cubelos B Nieto M. Enrichment of conserved synaptic activity-responsive element in neuronal genes predicts a coordinated response of MEF2, CREB and SRF. PLoS ONE. (2013) 8:e53848. 10.1371/journal.pone.0053848 23382855 PMC3561385

[56] Pulimood NS Rodrigues WDSJ Atkinson DA Mooney SM Medina AE. The Role of CREB, SRF, and MEF2 in Activity-Dependent Neuronal Plasticity in the Visual Cortex. J Neurosci. (2017) 37:6628–37. 10.1523/JNEUROSCI.0766-17.2017 28607167 PMC5508254

[57] Cole CJ Mercaldo V Restivo L Yiu AP Sekeres MJ Han JH. MEF2 negatively regulates learning-induced structural plasticity and memory formation. Nat Neurosci. (2012) 15:1255–64. 10.1038/nn.3189 22885849

[58] Flavell SW Greenberg ME. Signaling mechanisms linking neuronal activity to gene expression and plasticity of the nervous system. Annu Rev Neurosci. (2008) 31:563–90. 10.1146/annurev.neuro.31.060407.125631 18558867 PMC2728073

[59] Tai Y Feng S Ge R Du W Zhang X He Z. TRPC6 channels promote dendritic growth via the CaMKIV-CREB pathway. J Cell Sci. (2008) 121(Pt 14):2301–7. 10.1242/jcs.026906 18559891

[60] Flavell SW Cowan CW Kim TK Greer PL Lin Y Paradis S. Activity-dependent regulation of MEF2 transcription factors suppresses excitatory synapse number. Science. (2006) 311:1008–12. 10.1126/science.1122511 16484497

[61] Zhou J Du W Zhou K Tai Y Yao H Jia Y. Critical role of TRPC6 channels in the formation of excitatory synapses. Nat Neurosci. (2008) 11:741–3. 10.1038/nn.2127 18516035

[62] Wang J Zhang Q Chen Y Yu S Wu X Bao X. Novel MEF2C point mutations in Chinese patients with Rett (-like) syndrome or non-syndromic intellectual disability: insights into genotype-phenotype correlation. BMC Med Genet. (2018) 19:191. 10.1186/s12881-018-0699-1 30376817 PMC6208086

[63] D‘Haene E Bar-Yaacov R Bariah I Vantomme L Van Loo S Cobos FA. A neuronal enhancer network upstream of MEF2C is compromised in patients with Rett-like characteristics. Hum Mol Genet. (2019) 28:818–27. 10.1093/hmg/ddy393 30445463 PMC6381311

[64] Morrow EM Yoo SY Flavell SW Kim TK Lin Y Hill RS. Identifying autism loci and genes by tracing recent shared ancestry. Science. (2008) 321:218–23. 10.1126/science.1157657 18621663 PMC2586171

[65] Lipton SA Li H Zaremba JD McKercher SR Cui J Kang YJ. Autistic phenotype from MEF2C knockout cells. Science. (2009) 323:208. 10.1126/science.323.5911.208b 19131610

[66] Tu S Akhtar MW Escorihuela RM Amador-Arjona A Swarup V Parker J. NitroSynapsin therapy for a mouse MEF2C haploinsufficiency model of human autism. Nat Commun. (2017) 8:1488. 10.1038/s41467-017-01563-8 29133852 PMC5684358

[67] Zaslavsky K Zhang WB McCready FP Rodrigues DC Deneault E Loo C. SHANK2 mutations associated with autism spectrum disorder cause hyperconnectivity of human neurons. Nat Neurosci. (2019) 22:556–64. 10.1038/s41593-019-0365-8 30911184 PMC6475597

[68] Parker KJ Garner JP Libove RA Hyde SA Hornbeak KB Carson DS. Plasma oxytocin concentrations and OXTR polymorphisms predict social impairments in children with and without autism spectrum disorder. Proc Natl Acad Sci USA. (2014) 111:12258–63. 10.1073/pnas.1402236111 25092315 PMC4143031

[69] Husarova VM Lakatosova S Pivovarciova A Babinska K Bakos J Durdiakova J. Plasma oxytocin in children with autism and its correlations with behavioral parameters in children and parents. Psychiatry Investig. (2016) 13:174–83. 10.4306/pi.2016.13.2.174 27081377 PMC4823192

[70] Tomova A Husarova V Lakatosova S Bakos J Vlkova B Babinska K. Gastrointestinal microbiota in children with autism in Slovakia. Physiol Behav. (2015) 138:179–87. 10.1016/j.physbeh.2014.10.033 25446201

[71] Clark RI Tan SW Pean CB Roostalu U Vivancos V Bronda K. MEF2 is an *in vivo* immune-metabolic switch. Cell. (2013) 155:435–47. 10.1016/j.cell.2013.09.007 24075010 PMC3807682

[72] Sharon G Sampson TR Geschwind DH Mazmanian SK. The central nervous system and the gut microbiome. Cell. (2016) 167:915–32. 10.1016/j.cell.2016.10.027 27814521 PMC5127403

[73] Alaerts K Bernaerts S Vanaudenaerde B Daniels N Wenderoth N. Amygdala-hippocampal connectivity is associated with endogenous levels of oxytocin and can be altered by exogenously administered oxytocin in adults with autism. Biol Psychiatry Cogn Neurosci Neuroimaging. (2019) 4:655–63. 10.1016/j.bpsc.2019.01.008 30846366

[74] Havranek T Lestanova Z Mravec B Strbak V Bakos J Bacova Z. Oxytocin modulates expression of neuron and glial markers in the rat hippocampus. Folia Biol. (2017) 63:91–7. 28805558 10.14712/fb2017063030091

[75] Lestanova Z Puerta F Alanazi M Bacova Z Kiss A Castejon AM. Downregulation of oxytocin receptor decreases the length of projections stimulated by retinoic acid in the U-87MG cells. Neurochem Res. (2017) 42:1006–14. 10.1007/s11064-016-2133-4 27995495

[76] Bakos J Srancikova A Havranek T Bacova Z. Molecular mechanisms of oxytocin signaling at the synaptic connection. Neural Plast. (2018) 2018:4864107. 10.1155/2018/4864107 30057594 PMC6051047

[77] Zatkova M Reichova A Bacova Z Strbak V Kiss A Bakos J. Neurite outgrowth stimulated by oxytocin is modulated by inhibition of the calcium voltage-gated channels. Cell Mol Neurobiol. (2018) 38:371–8. 10.1007/s10571-017-0503-3 28493233 PMC11481833

[78] Zatkova M Reichova A Bacova Z Bakos J. Activation of the oxytocin receptor modulates the expression of synaptic adhesion molecules in a cell-specific manner. J Mol Neurosci. (2019) 68:171–80. 10.1007/s12031-019-01296-x 30888622

[79] Brusco J Haas K. Interactions between mitochondria and the transcription factor myocyte enhancer factor 2 (MEF2) regulate neuronal structural and functional plasticity and metaplasticity. J Physiol. (2015) 593:3471–81. 10.1113/jphysiol.2014.282459 25581818 PMC4560579

[80] Grinevich V Knobloch-Bollmann HS Eliava M Busnelli M Chini B. Assembling the puzzle: pathways of oxytocin signaling in the brain. Biol Psychiatry. (2016) 79:155–64. 10.1016/j.biopsych.2015.04.013 26001309

[81] Hasan MT Althammer F Silva da Gouveia M Goyon S Eliava M Lefevre A. A fear memory engram and its plasticity in the hypothalamic oxytocin system. Neuron. (2019) 103:133–146. 10.1016/j.neuron.2019.04.029 31104950

[82] Shalizi A Gaudilliere B Yuan Z Stegmuller J Shirogane T Ge Q. A calcium-regulated MEF2 sumoylation switch controls postsynaptic differentiation. Science. (2006) 311:1012–7. 10.1126/science.1122513 16484498

[83] Tsai NP Wilkerson JR Guo W Huber KM. FMRP-dependent Mdm2 dephosphorylation is required for MEF2-induced synapse elimination. Hum Mol Genet. (2017) 26:293–304. 10.1093/hmg/ddw386 28025327 PMC6075181

[84] She H Yang Q Shepherd K Smith Y Miller G Testa C. Direct regulation of complex I by mitochondrial MEF2D is disrupted in a mouse model of Parkinson disease and in human patients. J Clin Invest. (2011) 121:930–40. 10.1172/JCI43871 21393861 PMC3049386

[85] Flavell SW Kim TK Gray JM Harmin DA Hemberg M Hong EJ. Genome-wide analysis of MEF2 transcriptional program reveals synaptic target genes and neuronal activity-dependent polyadenylation site selection. Neuron. (2008) 60:1022–38. 10.1016/j.neuron.2008.11.029 19109909 PMC2630178

[86] Weisman O Zagoory-Sharon O Feldman R. Oxytocin administration alters HPA reactivity in the context of parent-infant interaction. Eur Neuropsychopharmacol. (2013) 23:1724–31. 10.1016/j.euroneuro.2013.06.006 23906646

[87] Hu J Qi S Becker B Luo L Gao S Gong Q. Oxytocin selectively facilitates learning with social feedback and increases activity and functional connectivity in emotional memory and reward processing regions. Hum Brain Mapp. (2015) 36:2132–46. 10.1002/hbm.22760 25664702 PMC6868957

